# Anterior Cervical Meningocele: Systematic Review of the Literature and Illustrative Case

**DOI:** 10.3390/jcm14217530

**Published:** 2025-10-24

**Authors:** Edoardo Ricci, Antonio Meola, Ilario Scali, Paolo Manganotti, Leonello Tacconi

**Affiliations:** 1 Neurology Unit, Department of Medicine, Surgery and Health Sciences, University Hospital and Health Services of Trieste—ASUGI, University of Trieste, Strada di Fiume, 447, 34149 Trieste, Italy; edoardo.ricci@asugi.sanita.fvg.it (E.R.); ilario.scali@asugi.sanita.fvg.it (I.S.); paolo.manganotti@asugi.sanita.fvg.it (P.M.); 2 Neurosurgery Unit, Department of Medicine, Surgery and Health Sciences, University Hospital and Health Services of Trieste—ASUGI, University of Trieste, Strada di Fiume, 447, 34149 Trieste, Italy; leonello.tacconi@asugi.sanita.fvg.it

**Keywords:** meningocele, cervical spine, cerebrospinal fluid, Klippel-Feil syndrome, neurofibromatosis

## Abstract

**Background/Objectives**: Anterior cervical meningocele (ACM) is a rare congenital condition characterized by the herniation of the meninges through a defect in the anterior vertebral column. ACM clinical management is not standardized because this condition is rare, and guidelines are missing. Hereby, a systematic literature review is performed to determine management options and outcomes. **Methods**: The case of a 62-year-old patient with incidental diagnosis of C3-C5 ACM is presented. A systematic review was conducted using standard PRISMA (preferred reporting items for systematic reviews and meta-analyses) guidelines for all cases of anterior cervical meningocele from 1837 to 2025. **Results**: The review provided nine clinical cases and our illustrative case. The median age was 47 years, with a predominance of female patients (70%). The most common presenting symptom was neck pain (60%), followed by paresthesia and hypoesthesia in the upper limbs. Four patients underwent conservative management with clinical and radiological follow-up, while four patients underwent neurosurgical intervention. Surgical treatment was complicated by cerebrospinal fluid (CSF) leak in two patients, and one of them developed meningitis. **Conclusions**: ACM is typically associated with mesodermal dysplasia and dural ectasia. ACM usually has a benign clinical course, requiring neurological follow-up and conservative management alone. However, a surgical approach should be considered in cases of vertebral instability or symptoms related to upper airway compression or upper gastrointestinal tract compression despite the high risk of CSF leak when surgical repair is attempted.

## 1. Introduction

Anterior meningocele is a rare congenital condition characterized by the herniation of the meninges through a defect in the anterior vertebral column [1]. This condition has been repeatedly documented in the sacral or thoracic regions [2,3,4], and it is most commonly identified at birth and accounts for approximately 10% of all cases of spina bifida [3]. Briant reported the first case of anterior meningocele in 1837, and since then, only 148 patients have been described. The embryologic origin of anterior meningocele remains unclear, though several hypotheses have been proposed [5]. Anterior meningocele is exceedingly rarer then posterior meningocele and has been associated with connective tissue disorders such as neurofibromatosis [1,6,7,8,9,10]. Neurofibromatosis type 1 (NF1) is a relatively common autosomal dominant disorder with highly variable clinical expression. Hallmark features include cutaneous signs such as café-au-lait macules, ocular findings like Lisch nodules, and a range of benign and malignant tumors, most notably neurofibromas [11]. Skeletal manifestations are also frequent, including scoliosis, sphenoid wing defects, and vertebral dysplasia [11]. The anatomical defects of anterior meningocele are thought to arise from a combination of mesodermal dysplasia and dural ectasia, which is typical of neurofibromatosis [1]. In this condition, prevertebral neurofibromas are relatively common in the cervical region and may become large enough to compromise the upper airway.

Moreover, mesodermal dysplasia results in bony defects, including enlarged intervertebral foramina and scalloped vertebral bodies, allowing the dysplastic meninges to herniate through these weakened bony structures. The etiopathogenesis of these lesions may involve several contributing factors, including trauma, cystic degeneration of a neurofibroma, and dural or osseous dysplasia.

Klippel–Feil syndrome (KFS) is another genetic condition associated with anterior cervical meningocele [10]. KFS, usually due to PAX or GDF6 genes alterations [12,13,14], exhibits marked phenotypic heterogeneity, most notably in the variable patterns of vertebral body fusion. Guille et al. proposed a three-type classification of KFS: type I consists of the en-block fusion of cervical and upper thoracic vertebrae; type II consists of the fusion of one or two cervical interspaces; and type III is combined cervical and lumbar fusions [13]. Although KFS is more commonly associated with malformations such as anterior myelomeningocele, resulting from mesodermal maldevelopment around the neural tube, a single case of anterior cervical meningocele in a patient with KFS has been reported [10].

The presence of an anterior cervical meningocele (ACM) in the high cervical region presents significant clinical challenges. According to the literature [1,6,7,8,9,10], this pathological condition is often asymptomatic and benign and therefore may not require surgical intervention. Nonetheless, the potential for progressive growth and associated complications makes its management particularly challenging. This includes the potential for torticollis and bone instability, which complicate both diagnosis and treatment planning.

In this report, we describe the case of a 62-year-old asymptomatic patient with an anterior cervical meningocele, along with associated bone erosion of the C3–C5 vertebrae in the absence of dysraphism. In addition, we provide a comprehensive systematic review of the literature, focusing on surgical and conservative approaches and clinical manifestations in patients with this rare condition.

## 2. Materials and Methods

### 2.1. Illustrative Case

**Figure 1:**
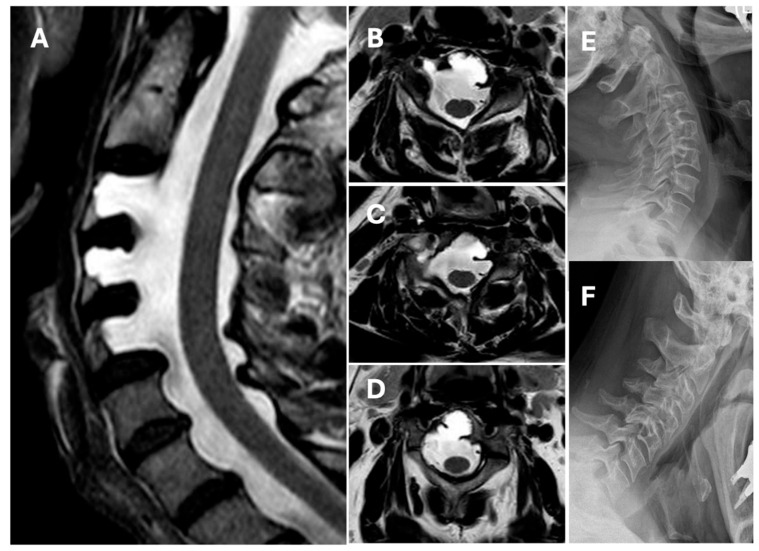
Illustrative case. (**A**) Magnetic resonance imaging (MRI) T2-weighted sagittal scan showing C3, C4, and C5 anterior cervical meningocele. (**B**) MRI T2-weighted axial scan showing C3 anterior meningocele. (**C**) MRI T2-weighted axial scan showing C4 anterior meningocele. (**D**) MRI T2-weighted axial scan showing C5 anterior meningocele. (**E**) Dynamic lateral X-ray scan of the neck in maximum extension and (**F**) in maximum flexion does not show signs of cervical instability.

We present the case of a 62-year-old male patient with no significant medical history who underwent ENT evaluation for nasal polyposis. A computed tomography (CT) scan of the cervical spine revealed large defects of the posterior wall of the vertebral bodies of C3, C4, and C5. The magnetic resonance imaging (MRI) scan revealed that the dural sac filled with cerebrospinal fluid occupied the defects. Post-contrast imaging did not reveal signs of bony rearrangement, vascular malformations, or tumors. CT head imaging did not reveal signs of lateral ventricle dilation, and the lumbar puncture performed showed no increase in cerebrospinal fluid pressure. Additionally, dynamic X-rays of the cervical spine in maximum flexion and extension did not reveal spinal instability (Figure 1). Genetic testing did not identify any mutations associated with either NF1 or KFS. Since there are no other known genetic conditions associated with ACM, further genetic testing was not recommended. A neurological evaluation found no focal deficits, while the patient denied pain.

### 2.2. Systematic Review of the Literature

**Figure 2:**
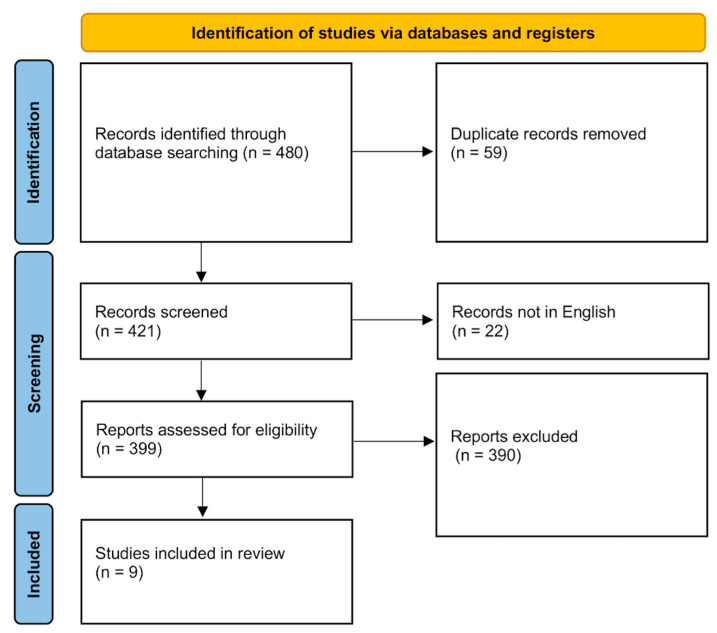
PRISMA (preferred reporting items for systematic reviews and meta-analyses) flowchart of systematic review for anterior cervical meningocele.

We conducted a systematic review following standard PRISMA (preferred reporting items for systematic reviews and meta-analyses) guidelines [15] to analyze all reported cases of cervical anterior meningocele without dysraphism from 1837 to 2025 (Figure 2 and Supplementary File S1). The systematic review is registered on Open Science Framework. A comprehensive search was performed in the PubMed, Cochrane Library, and Scopus databases using the keywords “Meningocele,” “Cervical,” and “Anterior.” This search yielded 480 studies, of which 59 duplicates were removed, leaving 421 studies for screening. The inclusion criteria were studies on anterior cervical meningocele. The exclusion criteria were studies on meningocele in locations other than cervical, post-traumatic cervical meningocele, and post-surgical meningocele; studies published in languages other than English; and studies without full text available. After full-text assessment, 390 studies were excluded and 9 were included in our review. We then extracted data on patients’ characteristics, ACM anatomical features, treatment modalities, and their outcomes. Given the small size of the cohort, only descriptive analysis was conducted.

### 2.3. Quality Assessment

The quality of each study was evaluated by a quality scale (Appendix A) based on JB Critical Appraisal Tools (JBI Manual for Evidence Synthesis. JBI, 2020. Available at https://synthesismanual.jbi.global; URL accessed on 1 March 2025). Among the others, there is a dedicated appraisal tool for case reports. Since all the papers included in the present review are case reports, the JB Critical Appraisal Tool for case reports was adopted for the present review. The overall quality scale consisted of eight questions, with “yes”, “no”, “unclear”, and “not applicable” as possible answers. Therefore, each paper was classified as “included” or “excluded” depending upon the total of “yes” answers. Specifically, any paper with at least five “yes” answers out of eight was included in the present review.

## 3. Results

### 3.1. Illustrative Case

At the six-month neurosurgical evaluation, the patient remained asymptomatic, with no objective signs of cervical instability. In the absence of clinical signs suggestive of spinal instability or fractures, surgical intervention was not indicated. A follow-up neurosurgical evaluation was recommended after six months, along with repeat MRIs of the cervical spine and dynamic X-ray scans.

### 3.2. Systematic Review of the Literature

All nine reports were clinical cases with a combined cohort of 10 patients, including our illustrative case (Table 1). The median age was 47 years, with a predominance of female patients (70%). The most common presenting symptom was neck pain (60%), followed by paresthesia and hypoesthesia in the upper limbs. Radiological evidence of esophageal or airway compression was noted in 30% of cases. Regarding underlying conditions, seven patients had NF1, while one case was associated with KFS. No syndromic association was reported in two cases. Four patients underwent conservative management with clinical and radiological follow-up, while four patients required neurosurgical intervention, including cyst excision and duraplasty (three cases), vertebral stabilization (one case), and hemilaminectomy (one case). In two cases, treatment details were not provided. The median follow-up was 6 months (IQR: 6-6). Follow-up data were available for six patients, of whom four remained asymptomatic. Two patients experienced cerebrospinal fluid (CSF) leak after surgical treatment, and one of them developed meningitis, too. Here, we present nine clinical cases identified in the systematic review:

1. A newborn female presented with an anterolateral cervical meningocele located in the posterior lateral triangle, causing torticollis and mass effect. Surgical excision was performed; however, the postoperative course was complicated by a cerebrospinal fluid fistula and subsequent meningitis [6].
2. A 51-year-old woman presented with motor deficit and paresthesia in the right upper limb, accompanied by pain triggered by neck movements. Cervical X-ray revealed multiple meningoceles. Physical examination identified multiple café-au-lait spots. Details regarding subsequent treatment and follow-up were not available [16].
3. A 40-year-old man with NF1 presented with neck pain accompanied by dysphagia. The pain radiated to the ipsilateral shoulder and was exacerbated by cough. Physical examination revealed soft tissue swelling in the neck and left upper limb weakness. Computed tomography myelography demonstrated multiple contrast-filled meningoceles in the prevertebral spaces. A conservative follow-up strategy was adopted, while neurosurgical intervention was not deemed necessary [1].
4. A 55-year-old woman with NF1 presented with worsening cervicothoracic pain of longstanding duration. Radiographs showed spinal canal enlargement with posterior scalloping at T6 and widened intervertebral foramina of C4–T1, predominantly on the right side. An MRI revealed multiple large anterior cervical meningoceles communicating with the dural sac through wide right-sided openings from C3–4 to the cervicothoracic junction, with minor dilations on the left. The cervical spinal cord was preserved without displacement. A myelogram and CT confirmed the findings. In the absence of neurological deficits, a conservative approach was adopted [7].
5. A 59-year-old woman with NF1 presented with progressive neck swelling, dysphagia, voice changes, and cervical pain. Physical examination revealed a 10 × 5 cm soft right-sided neck mass, enlarging on cough, multiple café-au-lait macules, cutaneous neurofibromas, and marked cervicothoracic kyphosis but no neurological deficits. CT and MRI showed a large fluid-filled anterior cervical mass, initially indistinguishable from cystic neurofibroma. A myelo-CT demonstrated thecal communication at the C3/4–C4/5 level, as confirmed by technetium-99 scintigraphy, consistent with an anterior cervical meningocele [17].
6. A 44-year-old man with NF1 presented with neck pain radiating to the left hand. Examination revealed cutaneous neurofibromas, mild weakness of the intrinsic hand muscles, and sensory loss in the C6–C8 dermatomes. Cervical radiographs demonstrated widening of the left C6–7 foramen, while CT myelography and MRI identified left-sided meningoceles at the C5–6 and C6–7 level, together with a suspected neuroma at C5–6. Due to persistent painful dysesthesia, the patient underwent a left C5 laminectomy, which revealed a neuroma arising from the posterior division of the C5 root and extending into the C5–6 foramen. The tumor was completely excised, and although the meningocele neck could not be repaired, it was wrapped with muscle and hemostatic material. The patient was discharged pain-free within one week, although sensory deficit in the C6–C8 dermatomes persisted. Histopathology confirmed neuroma. At the 6-month follow-up, an MRI showed stable meningoceles. Since the patient declined further intervention, no additional surgery was performed [8].
7. A 49-year-old man with NF1 presented with a 10-year history of globus sensation and progressive dysphagia, accompanied more recently by dysphonia, inspiratory stridor, and obstructive sleep apnea (OSAS). ENT evaluation revealed a large submucosal oropharyngeal–hypopharyngeal mass obstructing visualization of the larynx, along with polysomnographic evidence of severe OSAS. An MRI demonstrated three anterior cervical meningoceles (C3–6), the largest protruding through eroded foramina at C3–4 and C4–5, displacing the larynx, esophagus, and trachea. CT confirmed osseous dysplasia and foraminal erosion (C2–C5). Given the airway compromise, a tracheotomy was first performed, followed by a right anterolateral cervical approach. Two large meningoceles with surrounding neurofibromatous tissue were dissected, ligated, and excised, while a smaller intervertebral meningocele at C5–6 was left in place. The postoperative course was complicated by a cerebrospinal fluid leak, which required reoperation and lumbar drainage. The patient had an excellent recovery, with the complete resolution of dysphagia, airway obstruction, and OSAS and normal findings on follow-up laryngoscopy, videofluoroscopy, and polysomnography [9].
8. A 45-year-old woman with congenital fusion of C5–C7 presented after a fall and was investigated with an MRI, which demonstrated a cortical defect of the left C6–C7 through which an anterior cervical meningocele extended into the prevertebral space down to T1, as well as an associated syringomyelia from C4/5 to C7/T1. The cranio-cervical junction and the remainder of the spine and brain were normal, and laboratory studies were unremarkable. Neurological examination revealed no objective deficits, although the patient reported subjective upper limb weakness. There were no cutaneous or musculoskeletal abnormalities. She was conservatively managed with advice to avoid extreme cervical movements. At the 6-month follow-up, both her symptoms and MRI findings remained stable. She continues outpatient surveillance with serial imaging [10].
9. A 19-year-old woman with NF1 presented with long-standing torticollis, progressive bilateral visual loss over four months, and cerebellar symptoms for three months. Examination revealed early papilledema, positive bilateral cerebellar signs, and multiple café-au-lait macules and freckles on the trunk without evidence of symptoms attributable to the ACM. MRI of the brain demonstrated thickened optic nerves and a left cerebellar pilocytic astrocytoma with obstructive hydrocephalus, while cervical imaging revealed anterior herniation of the meninges into the retropharyngeal space at C2–C4, absent right lateral masses of C3–C4, hypoplastic right C2 pars and pedicle, and fusion of the C2–C3 posterior elements. Dynamic radiographs confirmed abnormal mobility at C3–C4 with atlanto-axial dislocation. The patient underwent suboccipital craniectomy with tumor excision, stabilization with bilateral lateral mass screws (C1, C3, and C4), and temporary prophylactic lumbar drainage. Postoperatively, she demonstrated complete tumor resection with the correction of torticollis. The patient underwent chemotherapy for optic glioma. At the 18-month follow-up, an MRI showed stable ACM size, although the patient remained asymptomatic [18].

### 3.3. Summary of Patients’ Outcome

Our review of the literature identified nine previously reported clinical cases, in addition to the case presented in this study. The following is a summary of the clinical outcomes according to the management approach adopted in each case:

- Surgical approach:
  1. Clinical outcome complicated by cerebrospinal fluid leakage and subsequent meningitis [6].
  2. Clinical outcome characterized solely by a persistent sensory deficit in the C6–C8 dermatomes [8].
  3. The clinical course was initially complicated by a cerebrospinal fluid leak requiring reoperation and lumbar drainage. At the six-month follow-up, the patient showed excellent recovery with no residual neurological deficits [9].
  4. The neurological status of the patient remained stable and was asymptomatic at final follow-up [18].
- Conservative approach:
  1. Follow-up not available [16];
  2. Follow-up not available [1];
  3. Follow-up not available [7];
  4. Follow-up not available [17];
  5. The neurological status of the patient remained stable and was asymptomatic at final follow-up [10];
  6. Our illustrative case: The neurological status of the patient remained stable and was asymptomatic at final follow-up.

### 3.4. Quality Assessment

All nine studies were “included”. Five studies [6,8,9,10,18] obtained a “yes” response to all eight questions of JBI Critical Appraisal. Four studies [1,6,7,17] received “no” as a response to one or more questions. Among these four papers, the most common questions with “no” as a response were, respectively, question n. 6: “Was the post-intervention clinical condition clearly described?”; question n. 7a: “Were adverse events (harms) or unanticipated events identified and described?”; and question n. 5: “Was the intervention (s) or treatment procedure (s) clearly described?” The highest number of “no” answers in a single paper was three [16,17].

## 4. Discussion

Spinal meningocele consists of the protrusion of the meninges through a defect of the vertebral bodies or foramina. Usually, spinal meningocele arises posteriorly to dysraphic vertebra as part of spina bifida presentation [19]. Anterior, antero-lateral, and lateral meningocele are much rarer and typically occur in the sacral and thoracic regions of the spine [20,21,22]. ACM is exceedingly rare, with only nine cases documented in the literature to date. ACM is usually associated with genetic abnormalities underlying NF1 and KFS. Mesodermal dysplasia and dural ectasia, which are characteristic features of NF1 and KFS, may contribute to vertebral body defects which, in turn, may facilitate anterior meningeal herniation [18]. However, the pathogenesis and natural history of ACM remain unclear, rendering therapeutic decision-making particularly challenging. Table 2 provides a summary of the main clinical characteristics, management strategies, and outcomes of the reported cases.

ACM is usually a manifestation of NF1. Out of nine patients reported in the literature, seven were affected with NF1 and one with KFS. NF1 is characterized by mesodermal dysplasia and dural ectasia, both of which may contribute to vertebral anomalies and structural weakening of the anterior dural sac. These alterations may predispose the development of ACM. An alternative etiopathogenetic hypothesis posits that dural ectasia leads to the enlargement of the intervertebral foramina, with subsequent cyst formation and erosion of the vertebral bodies. This mechanism may account for the occurrence of multiple anterior and anterolateral meningoceles in patients with NF1 [5]. Thus, given the association of ACM with syndromic conditions such as NF1 and KFS, genetic screening is recommended as part of the initial diagnostic workup. Surprisingly, the case reported here is a unique sporadic presentation of ACM in the absence of underlying genetic disorders. Thus, the alternative genetic mechanism underlying ACM should be considered.

In the reported cases, the median age at presentation is 47 years old (range: 41–54). The presented 62-year-old patient was therefore older than the median age reported in the literature. The most frequent presenting symptom is cervical pain, reported in six (60%) of the reviewed cases. However, both our patient and one previously described by Kos et al. [9] were asymptomatic at diagnosis. Symptoms attributable to mass effect, including dysphagia and upper airway compression, were observed in three cases (30%) [1,9,17]. Thus, in most cases, including the present case, the ACM diagnosis was incidental. Given the extreme rarity of ACM, guidelines about treatment and follow-up are missing. Diagnosis is usually based on CT and MRI scans. Dynamic X-rays in maximum flexion and extension of the cervical spine may be recommended to investigate the cervical stability of the spine. In the presence of cervical instability, spinal stabilization should be considered. Occasionally, myelo-CT and nuclear imaging may provide further proof of the presence and morphology of ACM.

A conservative approach with periodic radiological follow-up is commonly adopted in asymptomatic patients as the first choice of management. Conversely, if patients present signs of vertebral instability or mass effect, surgical treatment should be considered [6,8,9,18]. Typically, surgery consists of cyst excision with duraplasty and anterior cervical arthrodesis. In one case, posterior arthrodesis was performed because the patient underwent suboccipital craniotomy for resection of a tumor of the fourth ventricle [18]. In another case, posterior laminectomy was performed to resect a cervical schwannoma within the meningocele, although the approach was not intended to treat the meningocele [8]. Importantly, out of three cases treated with anterior cervical cyst resection, two were complicated by cerebrospinal fluid leak and one of them developed infective meningitis, too. Given the high risk of complications, surgical treatment should be reserved for selected cases.

## 5. Limitations

The main limitation of the present study is the retrospective nature of the included cases and the relatively short median follow-up of 6 months. Given the limited cohort size, a meta-analysis was not technically feasible. Moreover, while the immediate postoperative complications of ACM treatment are clear, the long-term natural history of ACM remains unknown, as well as the potential risk for cervical instability and spontaneous fracture if ACM is not treated. Thus, guidelines on the diagnosis and treatment of ACM cannot be generated. Finally, the genetic basis of ACM remains poorly understood, as also demonstrated by our case, which showed no association with NF1 or KFS. This limitation highlights the current gap in knowledge regarding the underlying genetic and pathological mechanisms, and future studies should aim to identify novel genetic alterations that may contribute to the development of this rare condition.

## 6. Conclusions

ACM is typically associated with NF1 or KFS and generally follows a benign clinical course requiring neurological follow-up and conservative management alone. Surgical intervention was generally reported in patients with vertebral instability or compressive symptoms, whereas asymptomatic cases were typically managed with clinical and radiological follow-up. However, the absence of long-term follow-up data in several cases limits our understanding of the clinical outcome of conservatively managed patients. Further research is needed to clarify the etiopathogenesis and natural history of ACM and to better define the indications for surgical and conservative management.

## Figures and Tables

**Table 1:** Overview of the reported cases of anterior cervical meningocele.

Author (Year)	N	Onset Symptoms	Age (yo)	Sex	Genetic Syndrome	Airway Comp.	Dysphagia	Level	Management	Follow-Up	Complications
Shore et al. (1982) [6]	1	Neck pain	Newborn	F	None	No	No	C2-C3	Cyst excision duraplasty	6 months	CSF leak and meningitis
O’Neil et al. (1983) [16]	1	Paresthesia	51	F	NF1	No	No	C4-C5	Conservative	N/A	N/A
Kaiser et al. (1986) [1]	1	Neck pain	40	M	NF1	No	Yes	C3	Conservative	N/A	N/A
So et al. (1989) [7]	1	Low backache	55	F	NF1	No	No	C3-T2	Conservative	N/A	N/A
Freund et al. (1992) [17]	1	Hypoesthesia C6-C8 and neck pain	59	F	NF1	Yes	Yes	C3-C5	Conservative	N/A	N/A
Göçer et al. (1999) [8]	1	Neck pain	44	M	NF1	No	No	C5-C7	Cyst excision duraplasty	6 months	None
Kos et al. (2009) [9]	1	Asymptomatic	49	F	NF1	Yes	Yes	C3-C6	Cyst excision duraplasty	6 months	CSF leak
Gallagher et al. (2015) [10]	1	Neck pain	45	F	KFS	No	No	C6-C7	Conservative	6 months	None
Kumar et al. (2021) [18]	1	Neck pain Headache	19	F	NF1	No	No	C2-C4	Posterior arthrodesis (C1-C4)	18 months	None
**Present case**	1	Asymptomatic	62	M	None	No	No	C3-C5	Conservative	6 months	None

**Abbreviations:** Airway comp.: airway compression; CSF: cerebrospinal fluid; KFS: Klippel–Feil syndrome; NF1: neurofibromatosis type 1; and N/A: not available.

**Table 2:** Systematic review of the reported cases of anterior cervical meningocele.

Variables	*n* = 10
**Demographic variables**	
Age (years)	47 (41–54)
Females [*n* (%)]	7 (70)
**Symptoms** [*n* (%)]	
Headache	1 (10)
Neck pain	6 (60)
Paresthesia/Hypoesthesia	2 (20)
Compressive signs	3 (30)
**Genetic syndrome** [*n* (%)]	
NF1	7 (70)
KFS	1 (10)
**Treatment** [*n* (%)]	
Conservative	4 (40)
Excision	3 (30)
Rod fixation	1 (10)
Hemilaminectomy	1 (10)
Not available	1 (10)
**Clinical outcomes** [*n* (%)]	
Asymptomatic	4 (40)
CSF leak	2 (20)
Meningitis	1 (10)
Not available	4 (40)

## Data Availability

The data will only be made available from the corresponding author upon reasonable request.

## Supplementary Materials

The following supporting information can be downloaded at: https://www.mdpi.com/article/10.3390/jcm14217530/s1, Supplementary File S1. The PRISMA 2020 reporting checklist.

## Abbreviations

ACM	anterior cervical meningocele
NF1	neurofibromatosis type 1
CT	computed tomography
MRI	magnetic resonance imaging
N/A	not available
KFS	Klippel–Feil syndrome
CSF	cerebrospinal fluid.

## Appendix A

**Table A1:** Quality assessment scores based on JB Critical Appraisal Tools (JBI Manual for Evidence Synthesis. JBI, 2020).

	Q1	Q2	Q3	Q4	Q5	Q6	Q7	Q8
Shore et al. (1982) [6]	Y	Y	Y	Y	Y	Y	Y	Y
O’Neil et al. (1983) [16]	Y	Y	Y	Y	N/A	N/A	N/A	Y
Kaiser et al. (1986) [1]	Y	Y	Y	Y	Y	N/A	N/A	Y
So et al. (1989) [7]	Y	Y	Y	N	Y	N/A	N/A	Y
Freund et al. (1992) [17]	Y	Y	Y	Y	N/A	N/A	N/A	Y
Göçer et al. (1999) [8]	Y	Y	Y	Y	Y	Y	Y	Y
Kos et al. (2009) [9]	Y	Y	Y	Y	Y	Y	Y	Y
Gallagher et al. (2015) [10]	Y	Y	Y	Y	Y	Y	Y	Y
Kumar et al. (2021) [18]	Y	Y	Y	Y	Y	Y	Y	Y
LEGEND								
Q1: Question 1, were patients’ demographic characteristics clearly described?								
Q2: Question 2, was the patient’s history clearly described and presented as a timeline?								
Q3: Question 3, was the current clinical condition of the patient on presentation clearly described?								
Q4: Question 4, were diagnostic tests or assessment methods and the results clearly described?								
Q5: Question 5, was the intervention(s) or treatment procedure(s) clearly described?								
Q6: Question 6, was the post-intervention clinical condition clearly described?								
Q7: Question 7, were adverse events (harms) or unanticipated events identified and described?								
Q8: Question 8, does the case report provide takeaway lessons?								
Y: yes; N: no; unclear; and N/A: not applicable.								
